# INTEGRATION OF PSYCHOLOGICAL ASSESSMENT SERVICES IN VHA COMMUNITY LIVING CENTERS

**DOI:** 10.1093/geroni/igae098.3435

**Published:** 2024-12-31

**Authors:** Kyle Page, HyeRim Ryu, Elizabeth MacDonald, Benjamin Szymanski, Kimberly Curyto, Michele Karel

**Affiliations:** Edward Hines, Jr. VA Hospital, Oak Park, Illinois, United States; Edward Hines, Jr. VA Hospital, Oak Park, Illinois, United States; Office of Mental Health and Suicide Prevention, Department of Veteran Affairs, Ann Arbor, Michigan, United States; Office of Mental Health and Suicide Prevention, Department of Veterans Affairs, Ann Arbor, Michigan, United States; Western New York VA Healthcare System, Batavia, New York, United States; VA Central Office, Washington, District of Columbia, United States

## Abstract

Psychologists embedded in Veterans Health Administration (VHA) Community Living Centers (CLC), or nursing home care settings, serve a crucial role in providing neurocognitive, clinical capacity, suicide risk and other evaluation services for residents with medical, psychiatric, and cognitive comorbidities. An anonymous national survey of CLC mental health professionals aimed to characterize the status of CLC mental health practice, including psychological assessment services. Eighty-two percent of respondents were psychologists (n=88). The majority (92%) do not complete portions of the Minimum Data Set (MDS) assessment. Reported practice varied regarding use of brief cognitive tests, with 57% using these tests when a resident they are following exhibits dementia warning signs. Focused or expanded cognitive testing batteries were conducted by the majority (75%), with 26% of respondents conducting them at least monthly. Eighty-seven percent of respondents reported that their cognitive evaluations had some to great impact on informing treatment plans. The large majority (94%) reported conducting medical decision-making evaluations at least sometimes, and at least half reported evaluating other decision-making or functional capacities. At the same time, a significant minority of psychologists reported that additional training regarding neurocognitive (27%) and capacity (46%) evaluations would be helpful. The majority (85%) indicated that, if the suicide risk screening upon admission to the CLC was positive, the psychologist typically completed a comprehensive suicide risk evaluation. Findings highlight the extent and importance of psychological assessment services to inform person-centered treatment planning for Veterans with complex care needs, pointing to areas of resource development to support these evaluation practices.

